# Pathogenicity and Genotyping of Fowl Adenovirus-D Serotype 2/11 Circulating in Commercial Broilers in Egypt

**DOI:** 10.3390/v18020252

**Published:** 2026-02-16

**Authors:** Eman Abd ElMenum Shosha, Ibrahim Eldaghayes, Saleh Esmate Ali Abdel-Rahaman, Amel Hussein, Heba M. El Naggar, Mohammed A. Gamaleldin, Ahmed Fotouh, Amina A. Radwan

**Affiliations:** 1 Virology Department, Faculty of Veterinary Medicine, New Valley University, El-Khargia 72511, Egypt; 2 Department of Microbiology and Parasitology, Faculty of Veterinary Medicine, University of Tripoli, Tripoli P.O. Box 13662, Libya; 3 Central Laboratory for Evaluation of Veterinary Biologics, Veterinary Serum and Vaccines Research Institute, Agriculture Research Center (ARC), Cairo 11381, Egypt; 4 Department of Poultry Disease, Animal Health Research Institute, Agricultural Research Center, Assiut 71526, Egypt; 5 Pathology and Clinical Pathology Department, Faculty of Veterinary Medicine, New Valley University, El-Khargia 72511, Egypt

**Keywords:** fowl adenoviruses, hexon gene, IBH-HPS, phylogenetic analysis, pathogenicity, Egypt

## Abstract

Fowl adenoviruses are opportunistic emerging viruses that spread widely in fowls, infecting birds of all ages, including young broiler chicks. This study aims to genotype the current adenovirus strains associated with inclusion body hepatitis hydropericardium syndrome (IBH-HPS) among infected broilers in Upper Egypt and to evaluate their pathogenic features. In 2024, 100 tissue samples were collected across Assiut and Sohag governorates in Upper Egypt for genetic characterization and pathogenicity evaluation. FAdVs were detected in 22% (11/50) of flocks. Typical FAdV lesions of dead embryos were observed after seven days post egg inoculation. Regarding the PCR assay of the hexon gene, only 8 of 30 samples were confirmed positive at 897 bp, yielding a 26.6% positivity rate. The remaining samples were considered negative using established RT-qPCR protocols for other viral pathogens. Partial sequencing of the hexon gene revealed that FAdV isolates (*n* = 4) clustered within FAdV species-D serotype 2/11, as determined by phylogenetic analysis. The four isolates shared (98–99%) and (94–100%) nucleotide and amino-acid similarities to FAdV-D of Israeli strains (2019–2020) and contemporary Egyptian isolates (2022), respectively, and low genetic divergence (54–81%) in comparison with other documented species. The amino acid sequence alignment and 3D structure indicate that the four immunogenic HVRs are located in the L1 region of the hexon protein, and that the highly conserved ^91^GQMTT^95^, a specific region for FAdV-D serotype 2/11, is present. Regarding pathogenicity, the gross and histopathological findings observed clearly demonstrate the systemic pathogenicity of FAdV-2/11 in the infected group, with a final mortality rate of 30% at seven days post-infection (dpi). The FAdV DNA in hepatic tissues and cloacal swabs was confirmed by the PCR method at 3 dpi and 5 dpi. These results emphasize the circulating of FAdV-2/11 species D in Upper Egypt and highlight the significant need for a single inactivated vaccine that effectively targets the relevant FAdV serotypes to achieve broader and more efficient protection.

## 1. Introduction

Over the past decade, various viral infections have become an increasingly major challenge, leading to multiple outbreaks with considerable effect on animal and poultry health [1,2,3,4,5,6]. Viral epidemics in poultry have been predominantly attributed to both RNA and DNA viruses, resulting in a significant threat and serious economic losses in the poultry populations [7,8,9,10]. Fowl adenoviruses (FAdVs) are opportunistic pathogens that widely spread in fowl flocks globally [11,12]. FAdVs are linear dsDNA, icosahedral, non-enveloped viruses that cluster in the genus Aviadenovirus, one member of the Adenoviridae family with a genome organization similar to that of other adenoviruses, isolated only from vertebrates across the animal kingdom [13]. There are five genera in the *Adenoviridae* family depending on the genome sequence of family viruses: Mastadenovirus, Aviadenovirus, Siadenovirus, Atadenovirus, and Ichtadenovirus [14,15]. FAdVs are avian viruses that infect birds of all ages, including young broiler chicks, and generally do not cause obvious clinical symptoms. FAdVs may be isolated from birds that show no clinical signs, as the birds carry the virus, and symptoms appear at different stages of infection. Interestingly, FAdVs have been detected in healthy poultry flocks, including those with high productivity and fertility [16,17]. The hexon protein gene is the major component of FAdVs, with its structure comprising two basement regions, P1 and P2, and the outer four loops, L1, L2, L3, and L4, which together comprise all seven hypervariable regions (HVRs) [18]. L1 loops exhibit the highest variation levels among HVRs. In addition, L1, L2, and L4 loops encompass antigenic and immunogenic segments that are used for typing and differentiation; in contrast, L3 lacks antigenic significance [19].

Clinically, infected birds have severe symptoms such as respiratory discomfort, uneven growth, arthritis, tenosynovitis, reduced egg production, enteritis, and different mortality rates [16]. Also, members of the genus Siadenovirus are responsible for clear hemorrhagic enteritis in turkeys and avian adenovirus, which causes splenomegaly predominantly in chickens, while members of the genus Atadenovirus can cause egg productivity drop syndrome. FAdVs belong to the genus Aviadenovirus, which has been identified and taxonomically divided into five species designated FAdV-A through FAdV-E. This classification is primarily based on molecular-structural criteria of the hexon gene, in combination with serological cross-neutralization tests [20]. Collectively, these species comprise 12 serotypes recognized, including (FAdV-1 to FAdV-8a, 8b, and FAdV-9 to FAdV-11). The two species D and E include FAdV-2, -3, -9, and -11 and FAdV-6, -7, -8a, and -8 b serotypes, respectively, which have a significant effect on poultry flocks [21,22]. Additionally, species A contains FAdV-1, species B comprises FAdV-5, and species C encompasses FAdV-10, -4 [20].

Postmortem signs associated with FAdVs infections in chickens are inclusion body hepatitis-hydropericardium pericardium (IBH-HPS) syndrome, hepatomegaly with friable texture and ecchymotic hemorrhages, as well as adenoviral gizzard erosion (AGE) [23,24]. IBH-HPS is a characteristic feature in broilers aged 3–7 weeks and in layers aged up to 20 weeks, with a subsequent decline in egg productivity [25]. In particular, FAdV-4 (species C) is virulent and causes fetal signs, making it more important than other FAdVs in the etiology of species causing IBH and HPS, leading to serious mortality [24,26]. FAdV-1/A produces AGE and is also associated with characteristic growth retardation of a large number of birds in the flocks [27,28].

Outbreaks of inclusion body hepatitis–hydropericardium syndrome have been reported in association with all FAdV serotypes in many countries globally; however, FAdV-IBH-HPS are frequently co-related to FAdV-D of FAdV-2/11 and FAdV-E, including FAdV-8a and -8b, causing serious economic consequences on the global poultry industry because of mortality rates ranging from 2–10%, and it may reach up to 70% concurrently with other co-infected immunosuppressive viruses. Besides, FAdVs’ immunosuppressive property and their growth retardation effects can lead to vaccination failure [22,29,30]. In Egypt, various species of FAdVs were identified in multiple epidemics [31]. FAdV-E of serotype 8a and FAdV-D 2/11 were first isolated from commercial broilers in Behira and Sharkia governorates during the period of 2019–2020 [28,32]. Moreover, the dominance of FAdV-D and FAdV-E, particularly serotype 8a, along with the emergence of recent FAdV serotypes (1, 3, and 8b), has been documented across the Delta governorates [17]. During 2021, the novel FAdV-C, a highly virulent species, was isolated in Alexandria governorate [33]. This study aims to identify the genotypes of the current adenovirus strains associated with IBH-HPS circulating among infected broilers in Upper Egypt. Additionally, it aims to investigate the pathogenicity of the current FAdV serotype to understand its pathogenic characteristics.

## 2. Materials and Methods

### 2.1. Ethical Declaration

All procedures, including animal handling and sampling in this research protocol, were performed according to the animal experiment ethical standards and approved by the Institutional Animal Care and Use Committee, Animal Health Research Institute, Agricultural Research Center, under the number Egypt (Approval No. ARC-AHRI-109-25), which complies with all relevant Egyptian legislations. The collected samples from the chicken farms were approved by the owners.

### 2.2. Study Period and Location

During 2024, 100 clinical specimens were collected from broiler flocks (*n* = 50) in various districts across the Assiut and Sohag governorates in Upper Egypt (Table 1). These selected governorates were based on postmortem reports of enlarged, friable livers with subcapsular ecchymotic hemorrhages with accumulated yellow fluid in the pericardial sac, elevated mortality, with suspicion of fowl adenovirus outbreaks in broilers.

### 2.3. Sampling and Specimen Preparation

Liver and spleen specimens were collected from freshly dead broilers with ages ranging from 1 to 33 days, as pooled samples, showing depression, body weight loss, and watery diarrhea. Specifically, the collected tissues from the same flock were combined to generate a single pooled sample per flock, yielding a total of 50 pooled samples representing 50 distinct flocks. The most prominent postmortem findings included pale, enlarged, friable livers with subcapsular ecchymotic hemorrhages, ascites, as well as accumulated yellow gelatinous fluid in the pericardial sac. The collected tissue specimens were pooled, homogenized in sterile phosphate-buffered saline (PBS; pH 7.4), containing 10% antibiotic solution, and incubated for 30 min. The homogenates were then clarified by centrifugation at 100× *g* and 4 °C for 10 min, and the resulting supernatant was stored at −80 °C until used for further analysis by virus isolation and PCR.

### 2.4. Isolation in Specific Pathogen-Free Embryonated Chicken Eggs (SPF-ECEs)

Inoculation of each processed supernatant (*n* = 50), 0.2 mL through the allantoic route of 9th-day-old SPF-ECEs (five ECEs per sample) [12] obtained from Koom Oshiem SPF poultry facility, Fayoum, Egypt, according to [34]. Eggs were incubated at 37 °C for 10 days, with continuous candling to record embryo viability. Embryos that had died after 24 h post-inoculation were culled, then the surviving ones were harvested for use in molecular detection by PCR.

### 2.5. Molecular Characterization of FAdVs

Using a QIAamp MinElute Spin Kit (Qiagen GmbH, Hilden, Germany), viral DNA was extracted according to the manufacturer’s protocol. The extracts were stored at −20 °C for further analysis. For the detection of FAdVs, a conventional polymerase chain reaction (PCR) was performed through oligonucleotide primers for the L1 region-hexon gene (*n* = 30) [sense primer *hexon* A: (5′-CAARTTCAGRCAGACGGT-3′) and antisense primer *hexon* B: (5′-TAGTGATGMCGSGACATCAT-3′)]. The expected product size was 897 bp in positions 144–161 and 1040–1120 for primer *hexon* A and B [35]. The PCR products were analyzed by 1.5% agarose gel electrophoresis (Applichem GmbH, Germany), then stained with 0.5 µg/mL ethidium bromide [36], and visualized and documented under UV transillumination.

### 2.6. Co-Infection Screening

All FAdV-D-field samples were subsequently screened for potential co-infection with other avian pathogens, including *Infectious bursal disease virus* (IBDV), Chicken *infectious anemia virus* (CIAV), *Newcastle disease virus* (NDV), *Avian leukosis virus* (ALV), *Infectious bronchitis virus* (IBV), and *Marek’s disease virus* (MDV), *Reticuloendotheliosis virus* (REV), and *Avian influenza virus* (AIV), using established RT-qPCR protocols. The corresponding primer and probe sequences (Table 2).

### 2.7. Sequencing and Phylogenetic Analysis of the L1 Region of the Hexon Gene

Using the QIAquick Gel Extraction Kit (QIAGEN, Hilden, Germany), the amplified 897 bp PCR amplicons were purified. For genetic evolution, the PCR amplicons (*n* = 4) of the hexon gene (L1 region) were bidirectionally sequenced by the Macrogen Clinical Laboratory using a Big Dye Terminator v3.1 Cycle Sequencing Kit (Applied Biosystems, Foster City, CA, USA) (South Korea) following the manufacturer’s instructions. The multiple-nucleotide sequences obtained for the four positive FAdVs isolates were deposited in GenBank under their accession numbers. The nucleotide and amino acid sequences were aligned with other FAdVs’ representative reference sequences of the hexon gene in the GenBank database, as well as vaccine lineages, using the Clustal W algorithm [37]. Phylogenetic analysis was performed using the maximum likelihood method, applying the Kimura 2-parameter model and 1000 bootstrap replicates, with MEGA version 7.0 and BioEdit software version 7.2.5 [38].

### 2.8. Antigenicity Prediction and Homology Modeling

To further characterize the hypervariable regions and identify mutations in the newly FAdV isolates, the three-dimensional (3D) structure of the L1 region-hexon protein was generated using the homology-modeling method in the automatic mode of SWISS-MODEL (Swiss Institute of Bioinformatics; https://swissmodel.expasy.org/, accessed on 1 October 2025). This approach allowed the investigation of homologous sequences and the construction of experimentally 3D protein models [39,40]. Subsequently, the resulting structures for representative isolates were visualized using PyMOL software (Version 1.7.4, http://www.pymol.org/), accessed on 1 January 2025 [41]. In silico prediction of potential antibody epitopes within the L1 region of FAdVs species-D was conducted using the IEDB Analysis Resource via a semi-empirical approach (http://tools.iedb.org/main/) accessed on 1 January 2025 [42]. The predicted epitopes exceeding the threshold (values > 1) were highlighted in yellow. Structural mapping of predicted epitopes was performed using the crystal structure of the CELO virus hexon protein (PDB ID: 2INY, chain A; 2.9 Å resolution), with analysis restricted to the L1 loop region corresponding to the amplified hexon fragment.

### 2.9. Virus Pathogenicity

The liver supernatants were inoculated into chicken embryo liver (CEL) cells for virus titer determination according to Reed and Muench [34], using the TCID50 method, and the collected supernatant was kept at −40 °C. The one-day-old SPF chicks (*n* = 100) purchased from Kom-Osheim farm, Fayoum, Egypt, were randomly divided into two groups (30 chicks in the control group and 70 in the infected group) under strict sanitary conditions. The use and care of animals in this research was conducted in accordance with OIE standards and ARRIVE guidelines to study the pathogenicity of FAdVs. The infected group was challenged with 0.2 mL/chick of virus solution containing 10^6^ TCID_50_/mL by intramuscular (I/M) injection in the chest region at 17 days of age. The control group received 0.2 mL of phosphate buffer saline. In each group, the clinical signs, mortality, and body weight (for growth rates assessment) were observed daily for 14 consecutive days following the challenge with the FAdVs isolate species D, serotype 2/11, Assuit-FAdV-D-1-hexongene-2024 (GenBank accession no.: PX254701). Three birds per group (infected and control) were randomly sacrificed for necropsy on days 1, 3, 5, 7, 9, 11, and 14 post-infection (dpi) to assess gross pathological lesions [43]. Finally, liver tissues and cloacal swabs were collected from three birds per group at each time point, processed as pooled samples prior to DNA extraction, and preserved at −20 °C for conventional PCR-based viral DNA detection. Extraction of viral DNA was performed using a QIAamp MinElute Spin Kit (Qiagen GmbH, Germany) according to the manufacturer’s guidelines. The target DNA sequence of FAdVs was amplified using the same PCR protocol and specific primers previously described [35]. Representative tissue samples from the liver, spleen, heart, and kidneys of birds in both the control and infected groups were collected. Each bird was humanely euthanized, and the selected organs were carefully excised to avoid mechanical damage. Tissue specimens measuring approximately 0.5–1 cm in thickness were immediately fixed in 10% neutral buffered formalin for a minimum of 24–48 h to ensure proper fixation. After fixation, samples were routinely processed, embedded in paraffin wax, and sectioned at 4–5 µm thickness using a rotary microtome. The sections were mounted on glass slides and stained with Hematoxylin and Eosin (H&E) for microscopic examination [44].

**Table 2:** Sets of consensus primer and probe applied for virus identification, subtyping, and sequencing in collected samples.

ID	Primer and Probe Sequences	Reference
AIV-M-gene	sep1: AGATGAGTCTTCTAACCGAGGTCG sep2: TGCAAAAACATCTTCAAGTCTCTG sep-probe: FAM-TCAGGCCCCCTCAAAGCCGA-TAMRA	[45]
IBV	AIBV-fr: ATGCTCAACCTTGTCCCTAGCA AIBV-as: TCAAACTGCGGATCATCACGT AIBV-TM: FAM-TTGGAAGTAGAGTGACGCCCAAACTTCA-TAMRA	[10,46]
NDV	F +4839: TCCGGAGGATACAAGGGTCT F −4939: AGCTGTTGCAACCCCAAG F +4894: FAM-AAGCGTTTCTGTCTCCTTCCTCCA-TAMRA	[47]
ALV	H5-F:GGATGAGGTGACTAAGAAAG H7-R: CGAACCAAAGGTAACACACG	[6]
IBDV	F/AUS GU: TCACCGTCCTCAGCTTACCCACATC R/AUS GL:GGATTTGGGATCAGCTCGAAGTTGC	[48]
CAV	CAV-F: GGTACGTATAGTGTGAGGC CAV-R: GCTGTGAGTGTTGCAAAGCT	[49]
REV	REV F: ACCTATGCCTCTTATTCCAC REV R: CTGATGCTTGCCTTCAAC	[50,51]
MDV	MDV-F: GGCACGGTACAGGTGTAAAGAG MDV-R: GCATAGACGATGTGCTGCTGAG	[52]

## 3. Results

### 3.1. Clinical Findings and Mortality Incidence

Clinically, the diseased birds from Sohag and Assiut governorates were suffering from respiratory manifestations and watery diarrhea, resulting in mortality rates ranging from 3–15%. The macroscopic findings revealed inflamed, swollen, pale livers with subcapsular ecchymotic hemorrhages and accumulated straw-coloured, gelatinous fluid in the pericardial sac; ascites; inclusion body hepatitis–hydropericardium–pericardium syndrome; enlarged, hemorrhagic kidneys and spleens with necrotic patches; peritonitis; and tracheitis (Table 1).

### 3.2. Virus Isolation and Molecular Characterization

**Figure 1:**
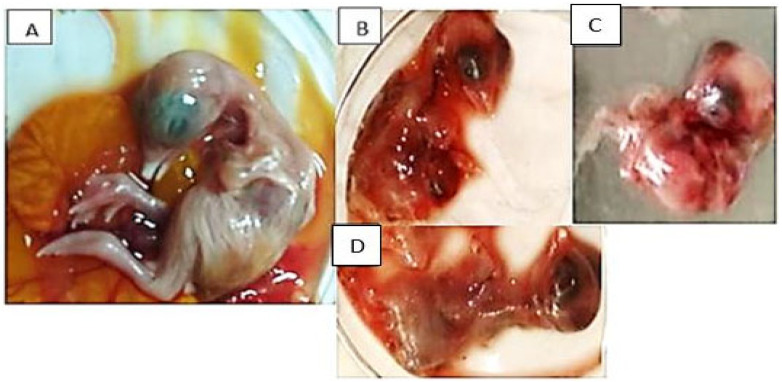
Macroscopic lesions of isolated FAdVs on SPF-ECE in comparison with a normal egg embryo. (**A**) Normal control embryo on the left side. (**B**–**D**) The dead embryos infected with FAdVs on the right side, showing hemorrhage, stunting, curling, liver necrosis, and splenomegaly.

**Figure 2:**
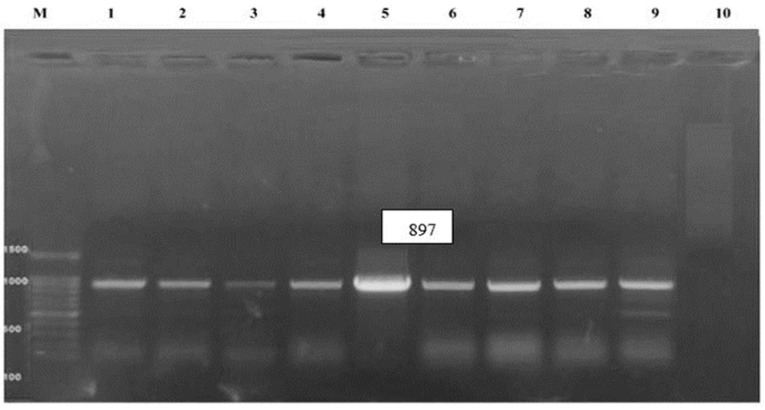
Identification of fowl adenovirus by PCR assay hexon gene (897 bp) for positive isolates using specific primers. Lanes 1–8 are positive samples, lanes 9 and 10 are positive and negative controls, respectively. Lane M represents a 100-bp ladder as a size standard.

Following 3–5 successful passages in the SPF-ECE, characteristic FAdV lesions were observed in the dead embryos seven days post-inoculation, including curling, stunting, subcutaneous hemorrhage, enlarged, fragile livers with yellowish necrotic spots, thickened chorioallantoic membrane (CAM), and splenomegaly (Figure 1). In contrast, using PCR analysis targeting the 897 bp L1 region of the hexon gene in the examined tissues, only 8 of 30 samples were confirmed positive, with a 26.6% individual-sample detection rate (Figure 2). The remaining samples were considered negative for other viral pathogens using established RT-qPCR protocols, including NDV, IBV, CIAV, ALV, REV, AIV, MDV, and IBDV, suggesting a FAdV infection without co-infection.

### 3.3. Sequencing and Phylogenetic Characterization

**Figure 3:**
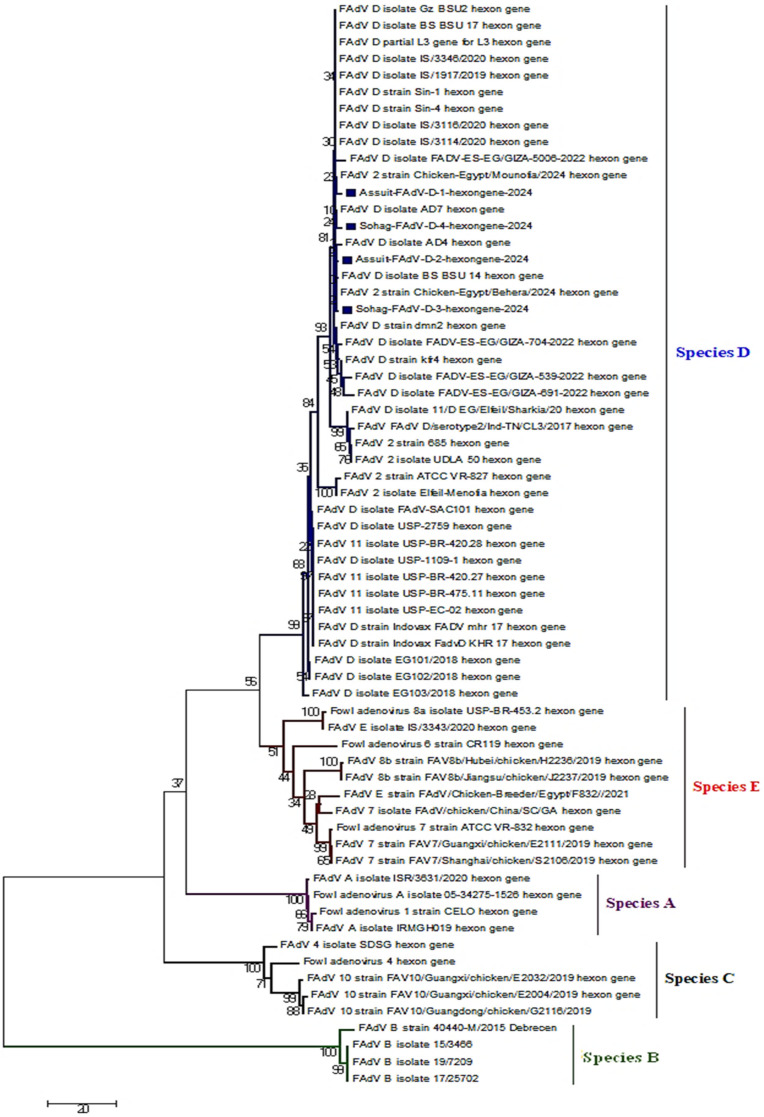
A phylogenetic tree was generated based on partial sequencing of hexon gene loop 1 (L1) region (897 bp) nucleotide sequences alignments of the studied isolates in comparison to reference and Egyptian Fowl adenovirus strains retrieved from GenBank. hexon L1 sequences representing all recognized FAdV species (A–E) were retrieved from the GenBank database. Reference sequences were selected based on sequence completeness, accurate species/serotype annotation, inclusion of recent Egyptian isolates, and representation of well-characterized international reference strains commonly used for FAdV classification. Phylogenetic analysis showed that four FAdV-D (serotype 2/11) isolates clustered together and are indicated by blue squares, while branch colors denote different FAdV species (A–E) as labeled on the right side of the tree. Sequence alignment was performed using the ClustalW algorithm, and the tree was designed using the Maximum Likelihood approach with 1000 bootstrap replicates in MEGA version 7.0, applying the general likelihood reversible substitution model. Bootstrap values ≥70% are shown at the corresponding nodes. The analysis demonstrates that the isolates sequenced cluster within the FAdV-D (serotype 2/11) clade, supporting their molecular classification based on partial hexon gene analysis.

**Figure 4:**
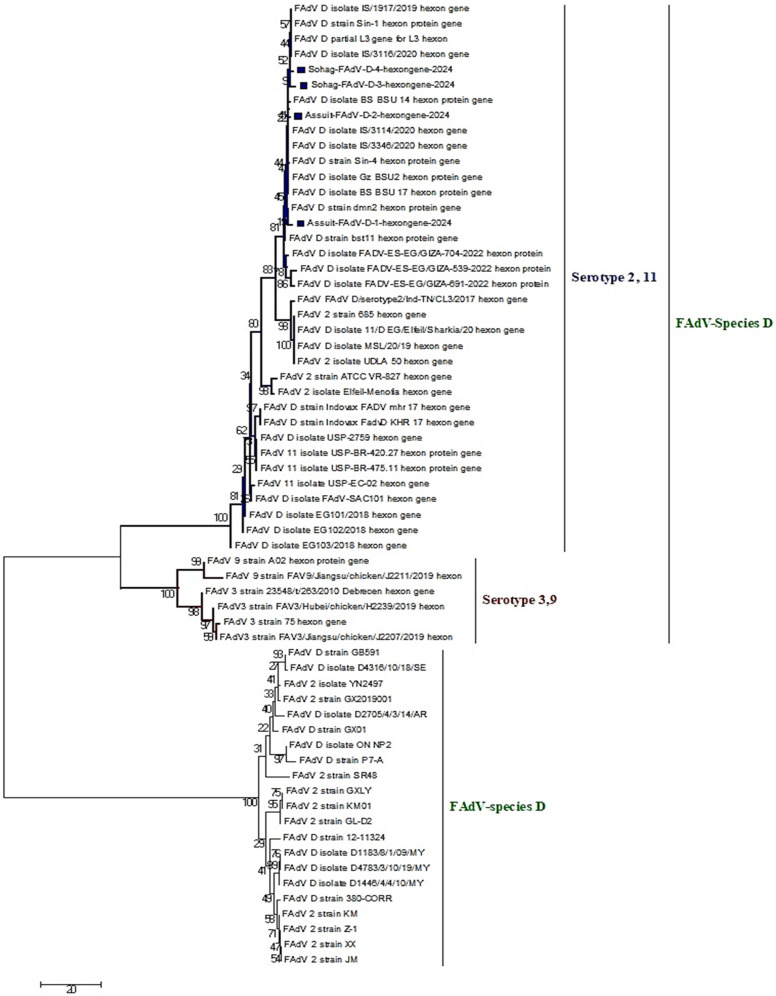
A phylogenetic tree of species D was generated based on partial hexon gene nucleotide sequence alignments (897 bp) of the studied FAdV-D isolates, compared with reference and Egyptian fowl adenovirus strains retrieved from GenBank. Phylogenetic analysis showed that four FAdV isolates clustered in species D, serotype2/11 and were indicated by blue squares. Additional clusters corresponding to serotype 3/9 (red branches) and other FAdV-D species (green branches) are shown. Bootstrap values ≥70% are shown at the corresponding nodes. The tree was constructed using the Maximum Likelihood approach with 1000 bootstrap replicates in MEGA version 7.0, under the general reversible substitution model.

Partial sequencing and phylogenetic tree of the L1 region-hexon gene from four representative isolates, Assuit-FAdV-D-1-hexongene-2024, Assuit-FAdV-D-2-hexongene-2024, Sohag-FAdV-D-3-hexongene-2024, and Sohag-FAdV-D-4-hexongene-2024 confirmed their classification as FAdV species-D serotype 2/11. The four sequences of isolates were deposited to GenBank, and their accession numbers were PX254701–PX254704 (Figure 3 and Figure 4). The four current sequences were compared and aligned with reference sequences from various FAdV species deposited in GenBank. Interestingly, our isolates, including Assuit-FAdV-D-1-hexongene-2024, Assuit-FAdV-D-2-hexongene-2024, Sohag-FAdV-D-3-hexongene-2024, and Sohag-FAdV-D-4-hexongene-2024 have the highest genetically related to FAdV species D, including MT127412: FAdV D isolate IS/1917/2019, MT759842: FAdV D isolate IS/3346/2020 (isolated in 2019–2020), OR400184: FAdV D isolate 2/11-ES-EG/GIZA-539-2022, OR400187: FAdV D isolate FADV-ES-EG/GIZA-704-2022 (Egyptian isolates) with nucleotide similarity of 94–99% and amino acid identity of 98–99%, respectively. Similarly, the four current isolates showed close genetic relatedness to strains circulating in the United Kingdom (KT862805: FAdV-2 strain 685) and MT975968: FAdV-D isolate EG101/2018 from Egypt, with 94–97% nucleotide and amino acid identities (Figure 3 and Figure 4). Similarly, the isolates exhibited 95–100% nucleotide and amino acid similarity with recent Egyptian isolates, including PP993160: FAdV D isolate Gz BSU2, PP993172: FAdV D isolate BS BSU14, and MZ355327: FAdV2 isolate Elfeil-Menofia (Egyptian isolates) (Table 3). In addition, the four isolates demonstrated nucleotide and amino acid homology (92–96%) with Brazilian, Saudi Arabian, and Pakistani strains, such as KY229175: FAdV 11 isolate USP-BR-420.28, MK995481: FAdV D isolate FAdV-SAC101, PV453994: FAdV D-11serotype strain; respectively. On the other hand, marked low genetic divergence (54–81%) was reported when compared with other species (A, E) and other serotypes (7, 8a, 8b) (Table 3). Notably, comparative sequence analysis between the recently sequenced isolates revealed 99–100% nucleotide and amino acid similarity to each other, indicating a close genetic relationship and likely common origin. Overall, the phylogenetic analysis based on the partial hexon L1 region showed that the Egyptian FAdV-D (serotype 2/11) isolates cluster with contemporary strains reported from Egypt and other regions, reflecting a high degree of genetic similarity within this genomic region and supporting their molecular classification.

Based on the partial hexon L1 region analysis, the Egyptian FAdV-D (serotype 2/11) isolates did not show close genetic relatedness to East Asian strains. Instead, they clustered predominantly with contemporary Egyptian, Middle Eastern, European, and some South American isolates, exhibiting higher nucleotide and amino acid identities within these lineages. The comparatively lower similarity observed with East Asian isolates suggests a limited recent viral exchange between East Asian and Egyptian FAdV-D populations, at least within the analyzed hexon region. These findings indicate that the circulating Egyptian FAdV-D strains likely evolved locally or through regional transmission rather than direct introduction from East Asia.

### 3.4. Mutation and Homology Modeling Analyses

**Figure 5:**
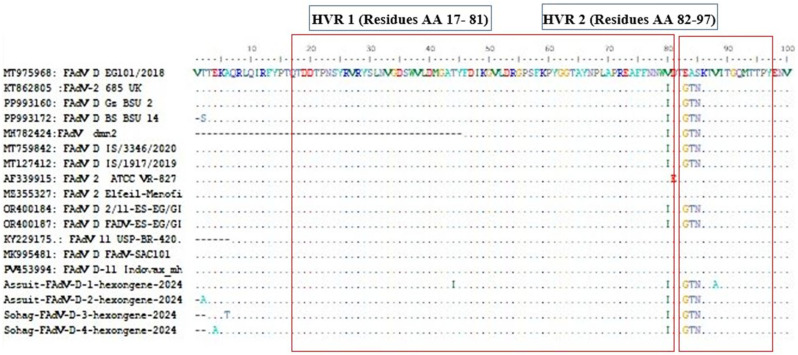
Multiple sequence alignment of the L1 region of the hexon gene from FAdV-D isolates and reference strains, including Egyptian isolates, highlighting hypervariable regions (HVRs 1–2) and predicted antigenic peptides (indicated by red boxes) according to [18]. The letters denote amino acid substitutions, while dots (.) represent identical amino acids, and dashes (−) indicate alignment.

**Figure 6:**
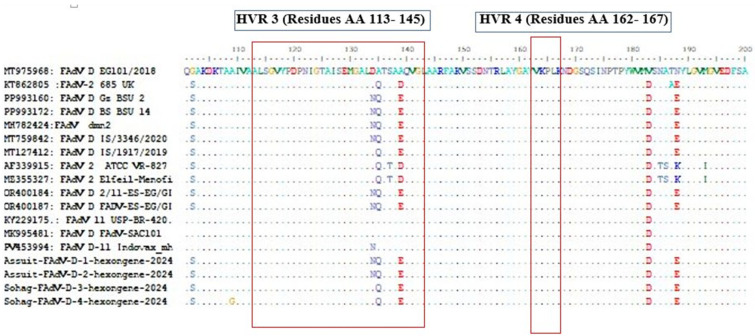
Multiple sequence alignment of the L1 region of the hexon gene from FAdV-D isolates and reference strains, including Egyptian isolates, highlighting hypervariable regions (HVRs 3–4) and predicted antigenic peptides (indicated by red boxes) according to [18]. The letters denote amino acid substitutions, while dots (.) represent identical amino acids, and dashes (−) indicate alignment.

**Figure 7:**
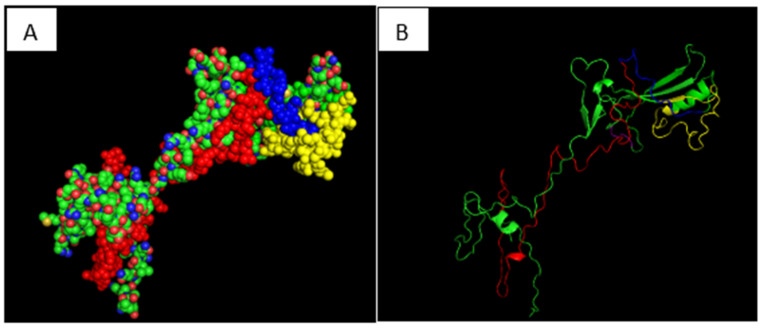
Tertiary structure modeling of the L1 region of the hexon protein in Fadvs-D, highlighting HVRs and conserved regions. (**A**) The 3D structure of hypervariable regions 1–4 within the L1 region. HVR1 is shown in red, HVR2 in blue, HVR3 in yellow, HVR4 in purple, and the conserved species-specific region in green. (**B**) lateral view of L1 region illustrates four HVRs.

The aligned amino acid (aa) sequences of the L1 region-hexon protein revealed different mutations and drifts in its amino acid sequence in the four HVRs regions, including HVR1: aa17–81; HVR2: aa 82–97; HVR3: aa113–143; and HVR4: aa162–167) (Figure 5 and Figure 6). To precisely identify the four HVRs in the recently characterized FAdV-D isolates, a 3D model of the L1 region based on the hexon protein was designed using a homology-modeling approach, with the avian adenovirus CELO strain as the proposed model (template no. 2INY.1.A). All six predicted peptide epitopes (Table 4) were located within the structurally L1 loop of the hexon protein as defined by alignment with the CELO hexon template, supporting their accessibility as putative antibody-binding regions. The resulting model was analyzed and visualized using Chimera software version 1.16 to explore the evolutionary characteristics of the target protein. This protein structure identified and illustrated the locations of the four HVRs on the hexon protein surface using PyMOL (Version 1.7.4) (Figure 7).

### 3.5. Linear Antibody Epitope Prediction

In silico analysis revealed that the L1 region of FAdV-D contained varying numbers of predicted linear antibody epitopes (Table 4). A total of six peptide regions exceeded the prediction threshold and were therefore classified as computationally predicted epitopes. Variations in the predicted peptide sequences were observed among FAdV-2/11 isolates, particularly within the HVR3 and HVR4 regions. In addition, FAdV-2/11 of species D showed variations in the predicted antigenic peptides in HVR3 and HVR4. The four isolates also contained a highly conserved region ^91^GQMTT^95^. These findings are based on bioinformatic prediction and represent preliminary hypotheses that require experimental validation to confirm their antigenic relevance.

### 3.6. Pathogenicity of the Isolated Virus

**Figure 8:**
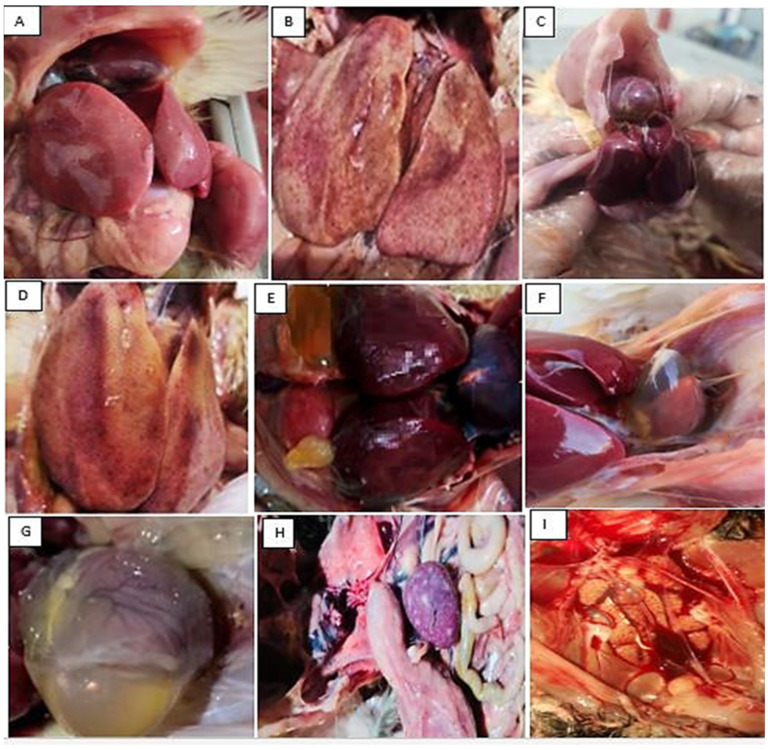
Typical gross pathology of infected birds with FAdVS serotype 2/11. (**A**–**E**) The liver was enlarged and fragile, displaying a yellowish-brown discoloration with scattered gray necrotic foci and subcapsular ecchymotic hemorrhages. (**F**,**G**) Heart showing serous fluid accumulation in the pericardium. (**H**) The spleen exhibited mild to marked enlargement with multifocal to coalescing necrotic foci. (**I**) The kidney was swollen and pale, with pinpoint hemorrhages.

**Figure 9:**
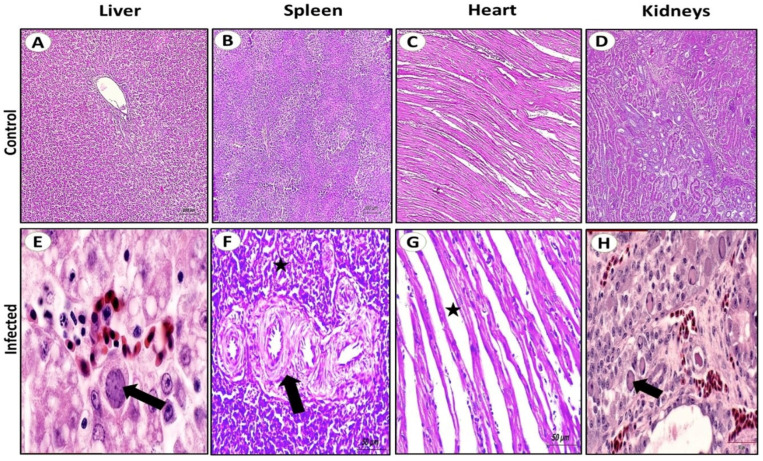
The photomicrograph of liver, spleen, heart, and kidneys from chickens in the control and infected groups (H&E stain). (**A**–**D**) Microscopic findings of the control group showed normal architecture for all examined organs (scale bar 200 μm). (**E**–**H**) The infected group (scale bar 50 μm) showed (**E**) Basophilic intranuclear inclusion bodies in hepatocytes (arrow). (**F**) The spleen showed lymphoid depletion in the white pulp (asterisks), and degeneration of splenic arterioles (arrow). (**G**) The heart exhibited interstitial edema (asterisks). (**H**) The kidneys showed intranuclear basophilic inclusions in tubular epithelium (arrow).

Birds infected with FAdV-2/11 exhibited symptoms including depression, ruffled feathers, and whitish or blood-stained droppings starting at 1 day post-infection (dpi). These manifestations progressively reduced by 9 dpi and completely resolved by 11 dpi. At necropsy, all birds in the control group exhibited clinically normal findings in the examined organs during the period of the experiment prior to death. The liver appeared normal in size, smooth, firm, and uniformly reddish-brown. The spleen was small, firm, and dark, with a sharp outline. The heart showed a normal shape and consistency, with smooth epicardial surfaces. The kidneys were of normal size and uniform dark tan coloration. The gross pathology at third dpi revealed that the most severe pathological lesions were in the liver. The liver appeared enlarged and fragile, displaying a yellowish-brown discoloration with scattered gray necrotic foci and subcapsular ecchymotic hemorrhages in most necropsied birds. At fifth dpi, the liver showed typical hepatitis, focal necrosis (white pinhead or red foci), a mosaic appearance with rounded edges, areas of hemorrhage, and hydropericardium with clear ascites, which persisted until the end of the experiment (Figure 8A–E). Also, the heart showed epicardial congestion, scattered petechial hemorrhages in the ventricles, and pericardial serous fluid accumulation (Figure 8F,G). The spleen frequently exhibited mild to marked enlargement (splenomegaly) with multifocal to coalescing necrotic foci scattered throughout the parenchyma (Figure 8H). The kidneys were typically swollen, pale, with accentuated lobulation and occasional pinpoint hemorrhages (Figure 8I). Concerning weight-gain assessment, the infected group gained 32 g throughout the experiment with no significant differences between the infected groups; meanwhile, the control group showed a mean weight gain of 71 g (Table 5). Death occurred at 7 dpi in the FAdV2/11 infected group, with a 30% mortality rate. Regarding viral DNA investigation, the genome was detected in liver tissues at 3 dpi by PCR and was monitored for 14 d until the end of the experiment; whereas in cloacal swabs, DNA was detected at 5 dpi and persisted until 11 dpi (Table 6). Histologically, all organs from the control group showed normal tissue architecture with no detectable pathological changes. The liver displayed intact hepatic cords, normal sinusoidal spaces, and hepatocytes with centrally positioned nuclei (Figure 9A). In contrast, the liver from the infected group exhibited multifocal to diffuse hepatocellular degeneration and coagulative necrosis, along with sinusoidal congestion and dense infiltration of mononuclear inflammatory cells. Many hepatocytes contained basophilic intranuclear inclusion bodies, which were characteristic of adenoviral infection (Figure 9E). The spleen of the control group presented well-organized white and red pulp with normal lymphoid follicular structure (Figure 9B). The infected group showed significant lymphoid depletion in the white pulp, focal necrosis of lymphoid follicles, and, in some sections, extramedullary hematopoiesis and degeneration of splenic arterioles (Figure 9F). The heart of the control group demonstrated normal myocardial fibers with clear striations and uniform nuclei (Figure 9C). The infected group showed some myocardial fiber fragmentation, along with interstitial edema and mild infiltration of mononuclear cells around blood vessels (Figure 9G). The kidneys of the control group had intact renal tubules and glomeruli with no signs of degeneration, congestion, or interstitial reaction (Figure 9D). The infected group exhibited tubular epithelial degeneration, vacuolation of renal tubules, and congestion of peritubular capillaries; occasional intranuclear basophilic inclusions were also observed in tubular epithelium (Figure 9H).

## 4. Discussion

Poultry health is impacted by a variety of avian viruses, many of which can be fatal, despite mass routine vaccination, which has had a negative effect on the local poultry farms in Egypt [50,53]. It is well known that FAdV can suppress the immune system, resulting in diminished humoral and cellular responses to other pathogens and vaccination regimens [54]. Lack of knowledge and the FAdV’s status as a primary pathogen in broilers may be contributing factors to worsening other infections and increased losses in the local poultry sector. FAdVs associated with IBH-HPS outbreaks in broilers are primarily species D (FAdV-2 and -11) and E (FAdV-8a and -8b), causing major economic losses globally [22]. There are many trustworthy diagnostic methods to investigate and identify FAdVs with IBH-HPS syndrome, including histopathology [55], and PCR assay [56]. Lately, FAdVs have received increasing attention worldwide as a severe infectious disease, with limited reports in Egypt. Thus, this study seeks to molecularly characterize field strains of FAdV linked to IBH-HPS circulating in Upper Egypt governorates, supplying surveillance data on genetic variability, and pathogenic properties in affected poultry populations.

In the current study, the detection of fowl adenovirus (FAdV) in 11 of 50 flocks (22%) reflects continued viral activity in Assiut and Sohag governorates and the commercial broilers, with a wide range of ages, are severely infected with FAdV. So, the viral infection was recorded in early-age chicks, suggesting vertical transmission from infected parental flocks or horizontal post-hatching. This notable finding was supported by [17,22,57], who reported FAdV early infection in susceptible chicks less than two weeks old. Clinically, the infected birds exhibited remarkable respiratory manifestations and watery diarrhea, with a 3–15% mortality rate, indicating that FAdVs were prevalent to varying degrees. Nevertheless, the significant mortality rate variations were also recorded in Japan, China, Canada, and Egypt [21,58]. Grossly, the necropsy findings revealed enlarged pale livers with subcapsular ecchymotic hemorrhages and straw-colored gelatinous fluid in the pericardium, causing a noticeable decrease in the heart size, ascites, IBH-HPS syndrome, enlarged hemorrhagic kidneys and spleens with necrotic patches. These findings were in line with previously reported clinical and post-mortem examinations [20,28,55,58,59,60,61,62]. Following SPF-ECE inoculation, dead embryos showed FAdVs typical pathognomonic lesions after 3–5 consecutive passages. These recorded findings were consistent with the observations of [12,61]. Also, these findings matched those of [22], who isolated the FAdV from one-day-old chicks, which may complicate disease control. The PCR confirmation was performed using primers designed to target the L1 region of the hexon gene, as this is the most hypervariable region and can distinguish between various FAdV species [19]. Furthermore, only eight samples tested RT-PCR-positive for the L1 region of the hexon gene (897 bp), with a 26.6% prevalence in broilers. Whereas, all specimens were negative for NDV, IBV, CIAV, ALV, REV, AIV, MDV, and IBDV, excluding mixed viral co-infection. This molecular pattern matches previous Egyptian findings of [12,58,61] and other reports documented by [17,19,63], who stated that the FAdV 2/11 species D was dominant in their studies. These findings demonstrate the continuous risk posed by FAdV during the production cycle. This is because the viral infection can persist in a latent or undetectable for a while before reactivation, possibly contributing to secondary bacterial and viral infections, especially in young or immunocompromised birds [64]. Moreover, the determination of FAdV-associated pathologic lesions and the viral genome at different ages indicates a broad age susceptibility [11,65]. In line with this, there are significant degenerative alterations in FAdV-infected chicks during their first week of life [66].

The phylogenetic analysis based on partial hexon gene sequencing revealed that FAdV isolates (*n* = 4) clustered within FAdV species-D serotype 2/11. The four isolates shared (98–99%) and (94–100%) nucleotide and amino-acid similarities to FAdV-D of Israeli strains (2019–2020) and contemporary Egyptian isolates (2022), respectively. Our current findings are consistent with [17,58,61]. Moreover, the close genetic relatedness observed between current Egyptian isolates and those reported from neighboring countries, such as Israel, may indicate regional genetic relatedness among circulating strains, potentially reflecting unregulated cross-border movement and smuggling across border areas. Likewise, the four isolates showed close homology, 94–97% nucleotide and amino acid identities with the reference British isolate (KT862805) and the reference Egyptian isolate (MT975968) detected during 2018–2022. The phylogeny results were consistent with [58,67], who reported similar nucleotide and amino acid homology of 94.2–97.9%. Comparative phylogeny analysis between the newly sequenced isolates showed extremely high nucleotide and amino acid identities (99–100%), indicating strong genetic relatedness and suggesting a common ancestral source. Taken together, these results indicate that the circulating strains in Upper Egypt cluster phylogenetically with previously reported Egyptian and international strains, reflecting substantial local circulation alongside broader transregional links. On the contrary, low genetic divergence (54–81%) was detected in comparison with other documented species (A and E) and serotypes (7, 8a, and 8b), may suggest a limited cross-protective immunity among these serotypes. This highlights the need for a single inactivated vaccine incorporating multiple serotype seed strains in Egypt to achieve broader and more efficient protection. The amino acid sequence alignment and 3D structure indicate that the four immunogenic HVRs are located in the L1 region of the hexon protein; this region is species-specific, with characteristic sequence lengths unique to each FAdV species [19,22]. The alignment of FAdV-D strains revealed ^91^GQMTT^95^, which is a highly conserved specific region for FAdV-D serotype 2/11. This conserved area is flanked by the hypervariable regions and is essential for maintaining the protein’s immunogenic and antigenic properties [68]. These observations agree practically with those findings documented by [17,58,61]. There is an amino acid substitution in the L1 region of the hexon protein, which can modify the amino acid encoding, consequently influencing the protein’s structural integrity and function, as well as the virus’s behavior.

The pathogenicity trial was conducted using one representative isolate (Assuit-FAdV-D-1-hexongene-2024) selected based on successful isolation, stable growth in CEL cells, and confirmed molecular identity as FAdV species D, serotype 2/11. This isolate was genetically representative of the circulating strains, as demonstrated by the high nucleotide and amino acid similarity among the four sequenced isolates. The mortality reached 30% at seven days post-infection in SPF chicks experimentally challenged with FAdV-D serotype 2/11. This rate is higher than the 3–15% mortality typically reported in field outbreaks, which can be attributed to the absence of maternally derived antibodies in SPF birds, as these antibodies provide partial protection in commercial flocks. Additionally, the experimental infection was performed via intramuscular inoculation with a defined high dose (10^6^ TCID_50_) of a single FAdV-D2/11 isolate, which may produce more uniform and severe disease than natural exposure. Regarding FAdV-2/11 pathogenicity, the gross and histopathological findings observed in this study clearly demonstrate its systemic pathogenicity in broiler chickens and strongly support previous reports on its organ preference and virulence [12,69]. The liver was the most severely affected organ with the highest viral load, which aligns with the well-known tropism of fowl adenovirus for hepatocytes [70]. Infected birds showed grossly pale, enlarged, friable livers. Similar liver changes have been documented by [43] in outbreaks of inclusion body hepatitis in broilers. The corresponding microscopic lesions, including hepatocellular degeneration, sinusoidal congestion, inflammatory infiltration, and, notably, basophilic intranuclear inclusion bodies, were characteristic signs of active viral replication. These inclusions indicate accumulations of viral proteins and nucleic acids within infected hepatocytes and have been consistently reported in both experimental and field cases of FAdV infection [12]. The spleen, a key immune organ, also exhibited characteristic lesions. Gross enlargement and congestion noted in infected birds match findings by [43], who observed that FAdV infection often causes splenomegaly due to increased antigenic stimulation and vascular changes. Microscopically, the spleen showed significant lymphoid depletion, reflecting a strong inhibitory effect on cell-mediated immunity. Lymphoid destruction is a common outcome of FAdV infection, likely due to viral replication in lymphoid cells or systemic cytokine imbalance [12]. This immunosuppression may make infected broilers more susceptible to secondary infections, increasing mortality and economic losses in affected flocks [70]. Cardiac lesions were less prominent than liver lesions but still noteworthy. Gross examination revealed epicardial congestion and hydropericardium, findings supported by [28], who showed that systemic FAdV infection can harm cardiac blood vessels. Microscopically, myocardial fiber interstitial edema suggests that the virus causes both direct cell damage and immune-driven inflammation. Such heart involvement can worsen systemic weakness and contribute to sudden death in broilers affected by FAdV. The kidneys also showed clear signs of viral damage. Grossly, swelling, pallor, and congestion of renal tissue were consistent with nephropathogenic effects described by [62]. Pathological changes included tubular epithelial degeneration and vacuolation with intranuclear inclusion bodies. These changes may result from direct viral replication in kidney tissue, as FAdV DNA has been detected in renal tissues in several studies [58]. Finally, control of FAdV infections is primarily achieved through vaccination; however, the single inactivated vaccine containing multiple serotypes and the decision to select a serotype vaccine remain challenging. Additional investigations are required to clarify the factors responsible for variations in pathogenicity among FAdV serotypes, which will support the adapted vaccines, ae well as understanding the disease pathogenesis.

## 5. Conclusions

This promising study provides pathogenic and molecular characterization of FAdV-2/11 of species D which confirms its circulating among commercial broilers in Upper Egypt during 2024. The infection was associated with clinical manifestations, mortality, and characteristic gross and histopathological lesions consistent with IBH-HPS. Genetic detection through PCR and SPF-ECE inoculation confirmed the viral presence, and sequencing of the hexon gene identified four representative isolates, sharing (98–99%), and (94–100%) nucleotide and amino-acid similarities to Israeli strains (2019–2020)**,** contemporary Egyptian isolates (2022), respectively. The observed low genetic similarity (54–81%) with other documented species (A and E) and serotypes (7, 8a, and 8b) highlights the significant need for a single inactivated vaccine that effectively targets the relevant FAdV serotypes, particularly in Upper Egypt, to achieve broader and more efficient protection. A major strength of this study is the combined use of clinical observations, molecular characterization, phylogenetic analysis, and viral pathogenicity, which together confirm the circulating of FAdV-2/11 strains in a region that remains poorly investigated. However, it should be noted that the pathogenicity assessment was based on a single viral isolate without biological replication, which limits the extent to which these findings can be generalized to all FAdV-D serotype 2/11 strains. Future investigations should prioritize whole-genome sequencing and biological replication, alongside antigenic mapping and experimental vaccine-matching approaches, to better inform national prevention and control strategies. In addition, further antigenic and immunological investigations—including cross-neutralization and vaccine-matching experiments—will be required to assess the implications of observed genetic diversity for vaccine design and control strategies. Overall, these findings highlight the continued circulation of FAdV-2/11 in species D in Upper Egypt, underscoring the pressing need for vaccine updates to strengthen FAdV control programs.

## Figures and Tables

**Table 1:** Summary of flock-level findings for FAdV detection in examined poultry flocks from the Upper Egypt governorates of Assiut and Sohag.

Sampling Governorate	Samples Number	Year	Type and Age of the Flock	Flock Size	Mortality %	Collected Samples		Signs and Postmortem Lesions Observed
Assiut	65	2024	Broilers, 1–33 days	5000–10,000	3–15	liver	spleen	Severe respiratory manifestations, depression, body weight loss, and watery diarrhea, enlarged, friable livers with subcapsular ecchymotic hemorrhages, accumulated yellow gelatinous fluid in the pericardial sac, ascites, hepatitis, hydropericardium, peritonitis, pneumonia, and tracheitis
						42	23	
Sohag	35		Broilers, 1–31 days	4000–9000	4–10	27	8	

**Table 3:** Comparative analysis of nucleotide and amino acid sequence identities of hexon gene-Loop 1 region among FAdV isolates and selected Egyptian and reference strains.

	Sequence	KT862805	MT975968	PP993160	PP993172	MH782424	MT127412	MT759842	AF339915	MZ355327	PV243152	OR400184	OR400187	KY229175	MK995481	PV453994	MW217576	ON502598	ON502594	KY229177	ON502594	Assuit-FAdV-D-1-hexongene-2024	Assuit-FAdV-D-2-hexongene-2024	Sohag-FAdV-D-3-hexongene-2024	Sohag-FAdV-D-4-hexongene-2024
Nucleotide identity %																									
1	KT862805: FAdV 2 strain 685	ID	95%	97%	97%	68%	98%	98%	96%	96%	82%	94%	94%	93%	96%	94%	63%	57%	65%	70%	64%	97%	97%	97%	97%
2	MT975968: FAdV D isolate EG101/2018	94%	ID	94%	94%	65%	95%	95%	95%	96%	79%	91%	91%	96%	99%	97%	63%	57%	64%	69%	63%	94%	94%	94%	94%
3	PP993160: FAdV D isolate Gz BSU 2	96%	95%	ID	99%	70%	99%	99%	95%	95%	83%	96%	97%	92%	95%	93%	64%	57%	66%	71%	65%	99%	99%	98%	98%
4	PP993172: FAdV D isolate BS BSU 14	96%	95%	98%	ID	70%	99%	99%	96%	95%	84%	95%	96%	93%	95%	94%	63%	57%	66%	70%	65%	99%	100%	99%	99%
5	MH782424:FAdV D strain dmn2	67%	65%	70%	70%	ID	69%	70%	67%	66%	83%	71%	72%	67%	66%	66%	43%	45%	53%	51%	52%	70%	70%	70%	70%
6	MT127412: FAdV D isolate IS/1917/2019	97%	96%	99%	99%	69%	ID	100%	96%	96%	84%	95%	96%	93%	96%	94%	63%	57%	65%	71%	64%	99%	99%	99%	99%
7	MT759842: FAdV D isolate IS/3346/2020	97%	96%	99%	99%	70%	100%	ID	96%	96%	84%	95%	96%	93%	95%	94%	63%	57%	65%	71%	64%	99%	99%	99%	99%
8	AF339915: FAdV 2 strain ATCC VR-827	95%	96%	95%	95%	65%	96%	96%	ID	99%	81%	92%	93%	94%	96%	95%	63%	58%	66%	71%	64%	95%	95%	95%	95%
9	MZ355327: FAdV 2 isolate Elfeil-Menofia	95%	96%	95%	95%	66%	96%	96%	100%	ID	80%	92%	92%	94%	97%	95%	63%	58%	66%	71%	64%	95%	95%	95%	95%
10	PV243152: FAdV 2 strain Chicken-Egypt/Mounofia/2024	81%	80%	82%	83%	83%	84%	84%	80%	80%	ID	79%	80%	81%	80%	80%	50%	52%	60%	57%	59%	83%	84%	84%	84%
11	OR400184: FAdV D isolate 2/11-ES-EG/GIZA-539-2022	92%	90%	96%	94%	71%	95%	95%	90%	91%	78%	ID	99%	89%	92%	91%	64%	59%	67%	73%	66%	96%	95%	94%	94%
12	OR400187: FAdV D isolate FADV-ES-EG/GIZA-704-2022	93%	92%	97%	95%	71%	96%	96%	92%	92%	79%	98%	ID	90%	92%	91%	64%	59%	68%	73%	67%	96%	95%	95%	95%
13	KY229175.: FAdV 11 isolate USP-BR-420.28	93%	97%	93%	94%	68%	95%	95%	95%	95%	83%	89%	90%	ID	97%	98%	62%	58%	67%	68%	66%	92%	93%	94%	94%
14	MK995481: FAdV D isolate FAdV-SAC101	95%	99%	96%	96%	66%	97%	97%	97%	97%	81%	92%	93%	98%	ID	98%	64%	57%	65%	70%	64%	95%	95%	95%	95%
15	PV453994: FAdV D-11serotype strain Indovax_mhr_17	94%	98%	95%	96%	67%	96%	96%	96%	96%	81%	91%	92%	98%	99%	ID	63%	57%	66%	70%	64%	94%	94%	95%	95%
16	MW217576: FAdV A isolate ISR/3631/2020	66%	66%	66%	65%	40%	66%	66%	66%	66%	50%	65%	66%	64%	66%	66%	ID	63%	63%	66%	61%	63%	63%	62%	62%
17	ON502598: FAdV 10/C FAV10/Guangxi/chicken/E2004/2019	54%	53%	55%	54%	40%	54%	54%	55%	55%	49%	54%	55%	55%	54%	54%	63%	ID	64%	60%	62%	57%	57%	57%	57%
18	ON502594: FAdV 7 strain FAV7/Shanghai/chicken/S2106/2019	75%	74%	76%	75%	59%	75%	75%	75%	75%	68%	75%	77%	77%	75%	76%	64%	59%	ID	81%	89%	66%	66%	66%	66%
19	KY229177: Fowl adenovirus 8a isolate USP-BR-453.2	81%	79%	82%	80%	57%	81%	81%	81%	81%	65%	82%	83%	78%	81%	80%	67%	58%	83%	ID	79%	71%	71%	70%	70%
20	ON502594: FAdV 8b strain FAV8b/Jiangsu/chicken/J2237/2019	73%	72%	74%	74%	57%	73%	73%	73%	73%	66%	74%	75%	75%	73%	73%	61%	59%	91%	81%	ID	65%	65%	65%	65%
21	Assuit-FAdV-D-1-hexongene-2024	96%	94%	99%	98%	69%	98%	99%	94%	95%	82%	94%	96%	93%	95%	95%	65%	54%	75%	81%	73%	ID	99%	98%	98%
22	Assuit-FAdV-D-2-hexongene-2024	96%	95%	98%	100%	70%	99%	99%	95%	95%	83%	94%	95%	94%	96%	96%	65%	54%	75%	80%	74%	98%	ID	99%	99%
23	Sohag-FAdV-D-3-hexongene-2024	96%	95%	97%	99%	70%	99%	99%	95%	95%	85%	93%	95%	96%	96%	96%	64%	55%	76%	80%	74%	97%	99%	ID	100%
24	Sohag-FAdV-D-4-hexongene-2024	96%	95%	97%	98%	69%	99%	98%	95%	95%	84%	93%	94%	95%	96%	96%	64%	54%	76%	79%	73%	97%	98%	99%	ID
Amino acid identity %																									

The nucleotide and amino acid similarities and divergences of the partially sequenced Loop 1 (L1) region of the hexon gene of the four Egyptian Fowl adenovirus isolates (indicated with grey color) were evaluated in comparison with selected global reference, field, and vaccine strains representing different FAdV species and serotypes. Comparative sequence alignment revealed high nucleotide identity (94–100%) and amino acid identity (98–100%) with FAdV species D serotype 2/11 strains, particularly recent Egyptian and international isolates. Moderate identities (92–96%) were observed with FAdV-D strains from Brazil, Saudi Arabia, and Pakistan. In contrast, marked sequence divergence was detected when compared with heterologous FAdV species (A and E) and serotypes (7, 8a, and 8b), with nucleotide and amino acid identities ranging from 57–81% and 54–81%, respectively.

**Table 4:** In silico–predicted putative linear antibody epitopes within the Loop 1 region of the hexon gene of FAdV species D identified using the IEDB analysis.

Species	No. of Predicted Peptides	Peptide
FAdV-D	6	^108^AAIVAALSGVYPD^120^
		^138^AAQVGLAARF^147^
		^98^AYGAYVKPL^106^
		^197^DFSASLTYPDTLLMP^211^
		^201^LTYPDTLLIPPPT^214^
		^239^INLLYHDTGVCSGT^252^

**Table 5:** Mean cumulative body-weight gain assessment (±SD) in the SPF chickens infected with FAdV2/11 over the experimental period (at different days post-infection).

Days Post-Infection (dpi)	Weight Gain (g) in the Infected Group (±SD) *	Weight Gain (g) in the Control Group (±SD)
0–1	2.2 ± 0.18	6.2 ± 0.22
1–3	6.1 ± 0.31	12.8 ± 0.27
3–5	12.4 ± 0.21	21.6 ± 0.33
5–7	19.3 ± 0.23	32.7 ± 0.25
7–9	25.2 ± 0.24	49.8 ± 0.16
9–11	28.1 ± 0.27	60.3 ± 0.14
11–14	32.3 ± 0.13	71 ± 0.24

* Values represent mean cumulative body-weight gain of surviving birds at each dpi. Standard deviation (±SD).

**Table 6:** PCR detection of FAdV DNA in liver tissues and cloacal swabs of experimentally infected birds at different time points post-infection.

Days Post-Infection							
Specimens	1	3	5	7	9	11	14
Liver	Negative	Positive	Positive	Positive	Positive	Positive	Positive
Cloacal swabs	Negative	Negative	Positive	Positive	Positive	Positive	Negative

At each time point, samples were collected from three birds per group and pooled prior to DNA extraction. “Positive” indicates detection of FAdV DNA by conventional PCR based on visualization of the specific amplicon at the expected size (897 bp). “Negative” indicates absence of detectable amplification under the applied PCR conditions.

## Data Availability

Data is available upon request.
